# HCl-based halide vapor phase epitaxy and selective-area HCl gas etching of (−112) β-Ga_2_O_3_

**DOI:** 10.1080/14686996.2026.2680969

**Published:** 2026-06-19

**Authors:** Takayoshi Oshima, Yuichi Oshima

**Affiliations:** Research Center for Electronic and Optical Materials, National Institute for Materials Science, Tsukuba, Japan

**Keywords:** β-Ga_2_O_3_, (−112), homoepitaxy, gas etching

## Abstract

We propose (−112) and crystallographically equivalent (1−1−2), (−1−12), and (11−2) planes as the fundamental crystal orientations for β-Ga_2_O_3_studies. These planes correspond to the {100} planes of the slightly distorted face-centered-cubic oxygen sublattice in β-Ga_2_O_3_and therefore represent one of the primary crystallographic planes. On this basis, we have demonstrated both homoepitaxial growth and HCl gas etching on (−112) β-Ga_2_O_3_substrates using an HCl-based halide vapor phase epitaxy system in order to clarify both the growth and etching characteristics on the (−112) plane. In homoepitaxy, the epilayer exhibited single crystallinity with tilt and twist dispersions comparable to those of the substrate and a step-and-terrace surface morphology with a root-mean-square roughness of 0.10–0.12 nm. Although slit-like pits whose sidewalls were vertically aligned (100) facets appeared on the surface, these pits were attributed to unintentional SiO_2_ nanomasks at the interface and are expected to be eliminated by improving pre-growth surface treatments or the initial growth process. Furthermore, the concentration of Cl impurities in the epilayer was as low as 1 × 10^15^ cm^−3^, which was significantly lower than 2 × 10^16^ cm^−3^ observed in the simultaneously grown (001)-oriented homoepitaxial layer. For HCl gas etching, selective-area etching was performed using a SiO_2_ mask with patterned windows. The etched structures clearly reflected the intrinsic crystal anisotropy. Side etching was minimized when the windows were aligned along the [02−1] direction due to the formation of exceptionally flat and vertical (100) facets, which possess the lowest surface energy density. Additionally, the vertical etch rate for the (−112) plane was approximately 50 times higher than the side etch rate for the (100) plane, enabling precise fabrication of high-aspect-ratio fins and trenches. These results–particularly the excellent surface smoothness achieved in homoepitaxy and the high-aspect-ratio patterning enabled by HCl-gas etching–demonstrate that the {−112} orientations are promising candidates for β-Ga_2_O_3_ studies.

## IMPACT STATEMENT

We demonstrate HCl-based halide vapor phase epitaxy and selective-area HCl gas etching on (−112)-oriented β-Ga_2_O_3_, enabling twin-free atomically flat homoepitaxy and high anisotropic etching with flat, vertical (100) sidewalls.

## GRAPHICAL ABSTRACT

**Figure:**
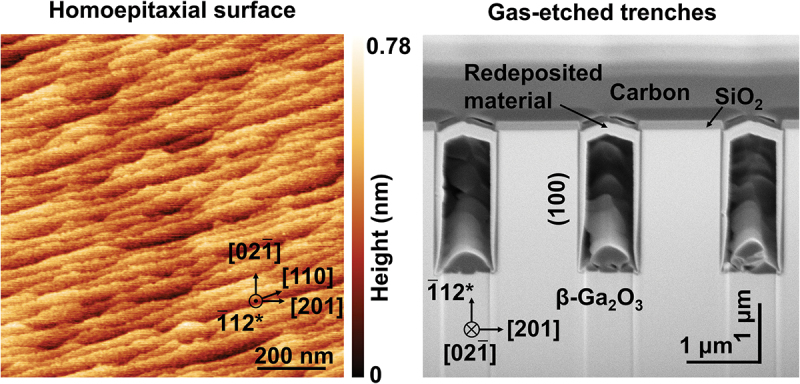

## Introduction

### β-Ga_2_O_3_ semiconductor

β-Ga_2_O_3_ has recently emerged as a promising ultra-wide-bandgap oxide semiconductor for next-generation power electronics applications [1]. This material offers compelling advantages over conventional non-oxide power semiconductors such as GaN, SiC, and diamond, including: (i) an exceptionally wide bandgap (≥4.43 eV) [2], which is among the largest for thermally stable binary metal oxides, enabling a high critical electric field of ~8 MV cm^−1^ [3]; (ii) high-speed, scalable bulk single crystal growth capabilities through established melt-growth techniques including floating zone [4], Czochralski [5], edge-defined film-fed growth [6], vertical Bridgman [7,8], and oxide crystal growth from cold crucible [9], with demonstrated wafer sizes up to 6 inches [1,8]; (iii) compatibility with diverse epitaxial growth techniques such as halide vapor phase epitaxy (HVPE) [10–16], metal-organic chemical vapor deposition (MOCVD) [17,18], mist chemical vapor deposition [19], molecular beam epitaxy [20–23], and pulsed laser deposition [24]; (iv) established bandgap engineering through alloying with Al_2_O_3_, Sc_2_O_3_, and In_2_O_3_ [25–30]; (v) potential for achieving high-quality metal-oxide-semiconductor (MOS) interfaces with low interface state densities due to SiO_2_/β-Ga_2_O_3_ heterointerfaces that can endure high temperature annealing [31–33]; and (vi) compatibility with ion implantation doping [34,35] and ion cutting techniques [36], facilitated by effective crystal lattice recovery through post-implantation thermal annealing. These features make β-Ga_2_O_3_ a highly attractive candidate for high-power, high-frequency electronic devices [1].

From a manufacturing perspective, high growth rate epitaxy is essential for realizing thick drift layers required for the most promising vertical power device applications, and HVPE is widely regarded as a promising technique for this purpose. However, achieving device-grade homoepitaxial layers requires not only a high growth rate but also high crystalline quality, low unintentional impurity incorporation, and smooth surface morphology compatible with subsequent device processing. In monoclinic β-Ga_2_O_3_, these properties are strongly orientation dependent; substrate orientation influences growth kinetics and defect manifestation and can lead to surface roughening [12] and twin formation [37,38]. Therefore, the exploration of substrate orientation remains a key issue for establishing a reliable β-Ga_2_O_3_ platform for high-voltage power devices.

### Exploring substrate orientations

To date, the research and development of β-Ga_2_O_3_-based vertical power devices have primarily relied on (001)-oriented substrates because the high HVPE growth rate on this orientation is well suited for preparing thick drift layers capable of sustaining high voltages for vertical power-electronic applications [10–14]. However, the surface morphology of the (001) epilayers is often rough due to the formation of groove-like pits associated with dislocations [12], which necessitates subsequent chemical mechanical polishing (CMP) to planarize the surface for device fabrication [39,40]. This post-epitaxy CMP increases manufacturing cost and is therefore preferably avoided. In this context, substrates with other orientations, which can yield smoother epitaxial surfaces, have attracted increasing attention.

The (100) orientation is a promising option for obtaining smooth epitaxial surfaces. Atomically flat epitaxial surfaces with well-defined step and terrace structures can be obtained owing to the lowest surface energy density of the (100) plane [20,41]. However, to suppress twin formation arising from double positioning of adatoms on the (100) surface, the substrate must be miscut by at least 6° from the (100) surface normal [37].

The (011) orientation is another attractive candidate for achieving flat epitaxial surfaces. Recently, Goto et al. demonstrated with Cl_2_-based HVPE (i.e. HVPE in which GaCl_*x*_ is produced by the reaction between Ga and Cl_2_) that the groove-like pits observed on (001) epilayers can be eliminated on (011)-oriented substrates, likely because the relevant dislocations lie parallel to the (011) plane [12]. Similar smooth (011) surfaces have been reported for HCl-based HVPE (i.e. HVPE in which GaCl_*x*_ is produced by the reaction between Ga and HCl) [15] and MOCVD [42]. However, the epitaxial surface was not atomically flat due to step bunching [15], suggesting that optimization of the off-angle optimization is required.

**Figure 1.**
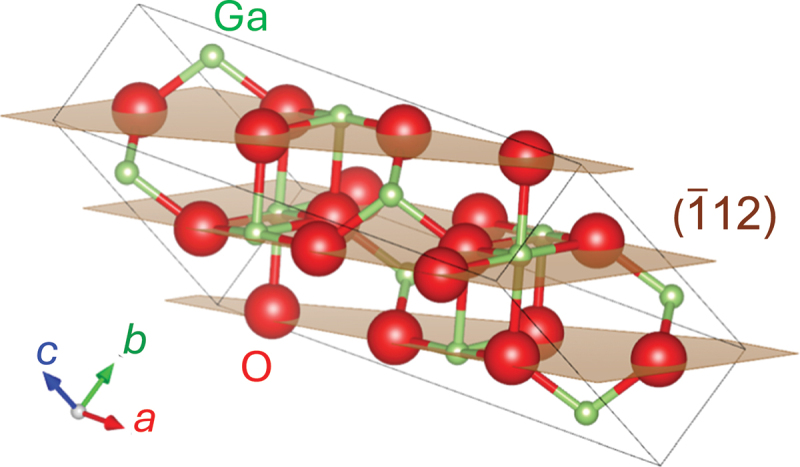
Unit cell of β-Ga_2_O_3_ showing the position of the ($\overset{ˉ}{1}$12) plane. The spacing between adjacent planes is 0.21 nm.

Thus, exploration of substrate orientations remains an open research area, and we introduce an oxygen-sublattice-based perspective to propose new crystal orientations. Although β-Ga_2_O_3_ crystallizes in a complex monoclinic structure, the fundamental framework is governed by a slightly distorted face-centered-cubic (fcc) oxygen sublattice, which allows a pseudo-cubic representation of the crystal structure and facilitates the exploration of substrate orientations [43]. Here, the lattice vectors of the non-primitive fcc-based unit cell are ***a***_**fcc**_ = ***b*** +1/2 ***c***, ***b***_**fcc**_ = − ***b*** +1/2 ***c***, and ***c***_**fcc**_ = 1/3 ***a*** +1/6 ***c***, which gives lattice parameters of *a*_fcc_ = *b*_fcc_ = 4.20 Å, *c*_fcc_ = 3.95 Å, *α*_fcc_ = *β*_fcc_ = 90.1°, and *γ*_fcc_ = 92.7°. In this fcc unit cell, the primary planes are {100}_fcc_, {110}_fcc_, and {111}_fcc_, which correspond to {100} and {$\overset{ˉ}{1}$12}; {010}, {$\overset{ˉ}{1}$02}, {512}, and {$\overset{ˉ}{7}$12}; and {$\overset{ˉ}{2}$01}, {101}, and {310} of the monoclinic unit cell [43]. Among these oxygen-sublattice-derived major planes, we chose ($\overset{ˉ}{1}$12)–or crystallographically equivalent (1$\overset{ˉ}{1}\overset{ˉ}{2})$, ($\overset{ˉ}{1}\overset{ˉ}{1}$2), or (11$\overset{ˉ}{2}$) planes–as a candidate orientation (see Figure 1 for the location of the ($\overset{ˉ}{1}$12) plane). One reason is that the ($\overset{ˉ}{1}$12) and (100) planes belong to the same {100}_fcc_ family [43], suggesting step-and-terrace epitaxial surfaces. Another reason is that the ($\overset{ˉ}{1}$12) plane is the closest to the (011) plane, with an interplanar angle of 19.7°, and is expected to yield groove-like-pit-free epitaxial surfaces.

The ($\overset{ˉ}{1}$12) orientation is also attractive from the viewpoint of crystallographic gas etching. We previously investigated HCl-gas etching on the most gas-etch-resistant (100)-oriented substrates and found that in-plane lateral etching was most pronounced along the direction perpendicular to the {$\overset{ˉ}{1}$12} planes [44]. This result indicates that the ($\overset{ˉ}{1}$12)-oriented substrates may enable the fastest vertical etching, potentially allowing the formation of extremely high-aspect-ratio trenches and fins with vertically aligned (100)-faceted sidewalls.

Therefore, in this study, we performed both homoepitaxial growth and gas etching on ($\overset{ˉ}{1}$12) β-Ga_2_O_3_ substrates to evaluate the potential of this orientation.

## HVPE on ($\overset{ˉ}{1}$12) β-Ga_2_O_3_

### Experimental methods

Homoepitaxy was performed on ($\overset{ˉ}{1}$12) and (001) β-Ga_2_O_3_ substrates using a custom-made HCl-based HVPE system that has been employed in our previous homoepitaxy experiments on (001), ($\overset{ˉ}{1}$02), and (011)-oriented β-Ga_2_O_3_ substrates [15,16]. This system is equipped with a horizontal quartz reactor that is divided into an upstream source zone and a downstream growth zone. A GaCl precursor was synthesized in the source zone at 610°C via the reaction between Ga metal (>99.99999% pure) and HCl gas (>99.999% pure). Under these conditions, the supplied HCl gas was completely consumed, generating an equimolar amount of GaCl gas. Homoepitaxy was carried out by introducing GaCl and O_2_ (>99.99995% pure) precursors, together with additional HCl gas to suppress parasitic reactions [45], onto the ($\overset{ˉ}{1}$12) and (001) β-Ga_2_O_3_ substrates mounted on a rotating holder in the growth zone heated at 1030°C. The reactive gases were carried by purified N_2_ gas (dew point < −110°C) under atmospheric pressure (approximately 100 kPa), and the total gas flow rate through the reactor was maintained at 8 slm. The partial pressures of GaCl, O_2_, and HCl were set at 0.125, 1.25, and 0.188 kPa, respectively. The growth time was 15 min.

The resulting epilayer was characterized using various methods. The epitaxial structure was examined using X-ray diffraction (XRD) measurements using Cu Kα_1_ radiation. The surface morphology was observed using differential interference contrast (DIC) optical microscopy, atomic force microscopy (AFM), and scanning electron microscopy (SEM). Cross-sectional observation was also performed using focused ion beam–SEM (FIB-SEM), scanning transmission electron microscopy (STEM) for high resolution energy-dispersive X-ray spectroscopy (EDS). The epilayer thickness and impurity concentrations of possible dopants (Si, Cl, N, and H) were measured by secondary ion mass spectrometry (SIMS) and the SIMS results were compared with those of the epilayer on the (001) substrate.

### Results and discussion

The growth rate on the ($\overset{ˉ}{1}$12) substrate was approximately half of that on the (001) substrate. The grown layer thickness and growth rate were 2.8 μm and 11 μm h^−1^ for the ($\overset{ˉ}{1}$12) orientation, and 6.1 μm and 24 μm h^−1^ for the (001) orientation, respectively. The ratio of the growth rate on ($\overset{ˉ}{1}$12) to that on (001) was 0.46, which is slightly lower than the previously reported growth-rate ratio of 0.6 for (011)/(001) in our previous study [15]. One possible reason for the lower growth rate on the $(\overset{ˉ}{1}$12) substrate than that on the (001) substrate is the lower surface energy density of the ($\overset{ˉ}{1}$12) plane, 62.4 meV Å^−2^, compared with that of the (001) plane, 87.9 meV Å^−2^ [46]. The lower surface energy density of the ($\overset{ˉ}{1}$12) plane suggests that this surface is relatively stable, with a lower density of dangling bonds or reactive bonding sites, thereby decreasing the incorporation probability of adatoms on the surface and resulting in a lower growth rate. For the (011) plane, we could not find literature reporting its surface energy density. However, the surface energy density of the (011) plane is expected to be higher than that of the ($\overset{ˉ}{1}$12) plane because the (011) plane is slightly inclined by 19.73° from the ($\overset{ˉ}{1}$12) plane that belongs to the {100}_fcc_ oxygen-sublattice plane, which likely results in a higher dangling-bond density. Therefore, it is also reasonable that the growth rate on the ($\overset{ˉ}{1}$12) plane is slightly lower than that on the (011) plane.

**Figure 2.**
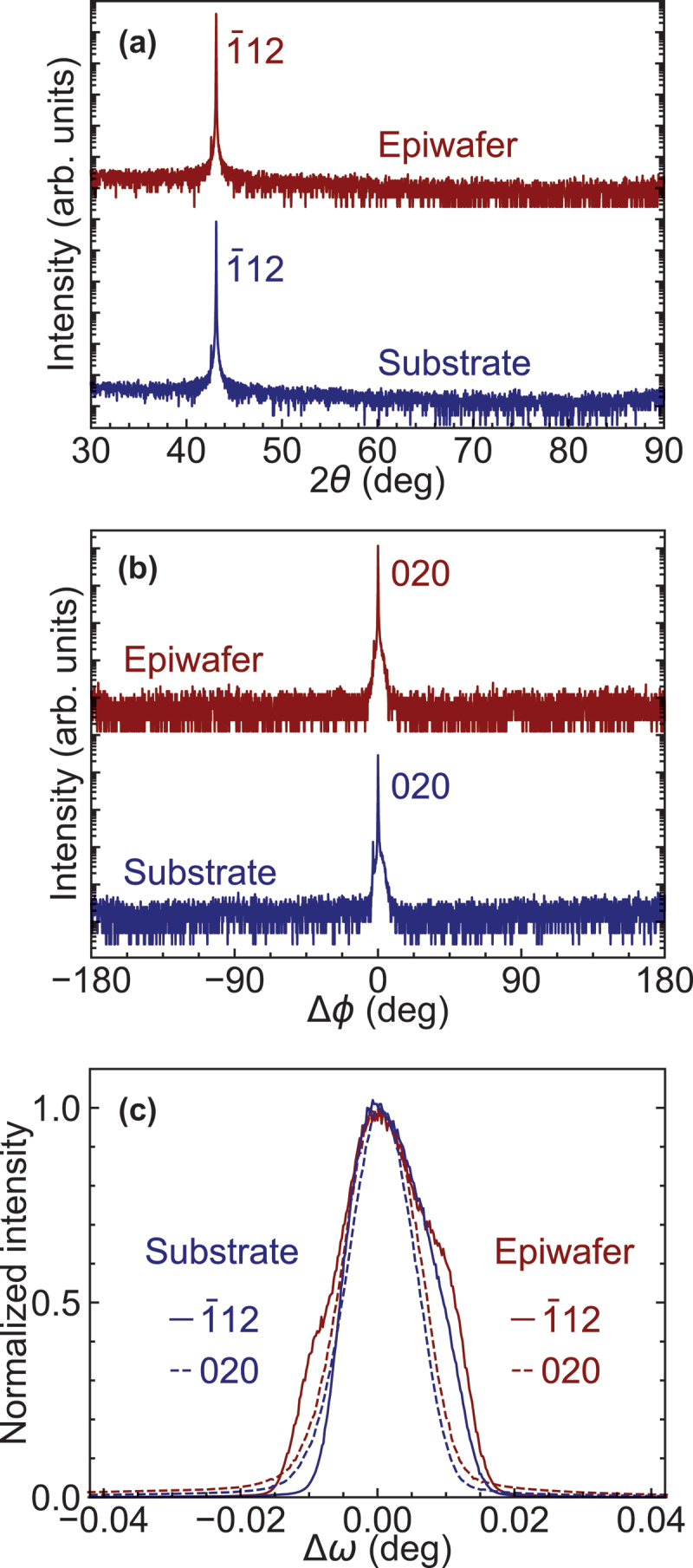
Summary of X-ray diffraction measurements of the ($\overset{ˉ}{1}$12) epiwafer and the corresponding substrate using Cu Kα_1_ radiation. (a) *θ*-2*θ* scan patterns for the $\overset{ˉ}{1}$12 diffraction. (b) Skew-symmetric *ϕ* scan patterns for the 020 diffraction. (c) *ω*-rocking curves of the observed $\overset{ˉ}{1}$12 and 020 diffraction peaks.

The ($\overset{ˉ}{1}$12) epitaxial layer exhibited single crystallinity, with tilt and twist dispersions comparable to those of the substrate. Figure 2 shows XRD patterns for the epitaxial wafer (epiwafer) and the corresponding substrate. In the *θ*-2*θ* patterns, only the $\overset{ˉ}{1}$12 diffraction peaks from the epiwafer were observed within the measured scan range at the same angles from the substrate (Figure 2(a)), indicating a single out-of-plane orientation without lattice strain in the layer. Note that the weak but sharp peak on the lower-angle shoulder of the main peak of the epiwafer originates from an impurity X-ray line other than Cu Kα_1_, and it also appeared in the substrate scan. In the skew-symmetric *ϕ* scan patterns, a strong 020 peak was observed for both the epiwafer and the substrate (Figure 2(a)), indicating the absence of rotation domains in the epilayer. The 020 peak was also accompanied by a weak but sharp shoulder peak in both scan patterns, which should be originated from the 021 diffraction because its reciprocal-lattice point lies close to that of 020. It should be noted that twin-free homoepitaxy was achieved on the ($\overset{ˉ}{1}$12) plane although both ($\overset{ˉ}{1}$12) and (100) planes belong to the same {100}_fcc_ family of the oxygen sublattice of β-Ga_2_O_3_, as described earlier. Figure 2(c) presents normalized *ω*-rocking curves of the $\overset{ˉ}{1}$12 and 020 peaks for both the epiwafer and the substrate. The full widths at half maximum (FWHMs) of the $\overset{ˉ}{1}$12 peak were 66 arcsec (epiwafer) and 55 arcsec (substrate), whereas those of 020 peak were 47 arcsec (epiwafer) and 42 arcsec (substrate), indicating that the epilayer maintains nearly the same tilt and twist spreads as the substrate and thus largely preserves the substrate crystalline quality.

**Figure 3.**
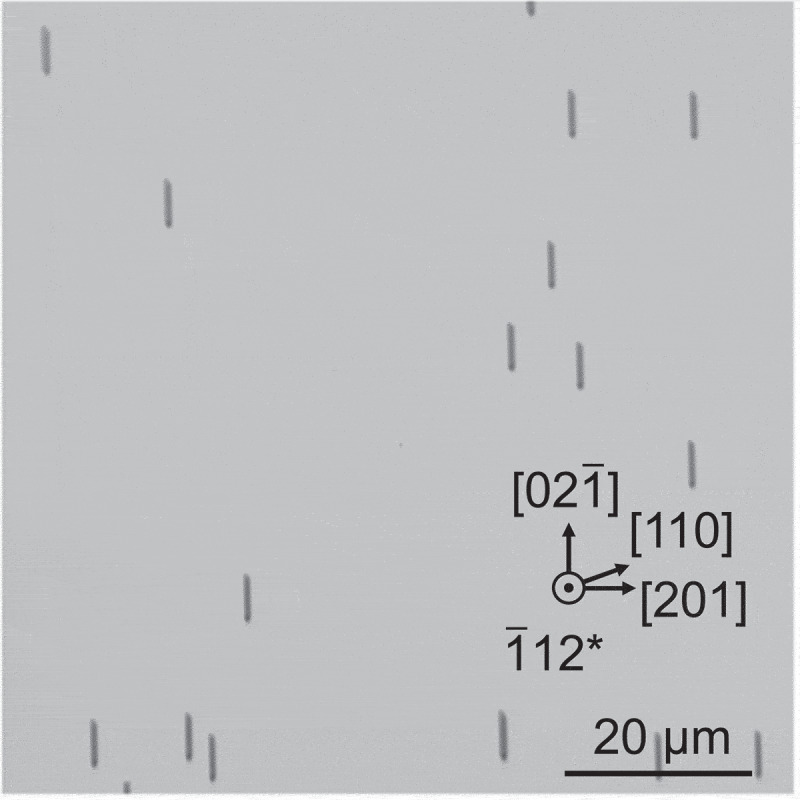
(a) Differential interference contrast optical microscopy image of the surface of the ($\overset{ˉ}{1}$12) epilayer.

The surface morphology of the ($\overset{ˉ}{1}$12) layer was very smooth overall, but some pits were present. Figure 3 shows a DIC optical micrograph of the homoepitaxial surface. Although groove-like pits reported for the (001) surface were not observed, numerous linear pits elongated along the $\left[02\overset{ˉ}{1}\right]$ direction were present, and the density of the pits was about 2 × 10^5^ cm^−2^. Notably, these pits had a nearly uniform length of approximately 5 μm, suggesting that they originated from the same depth. In contrast, the regions outside the pits were extremely flat and appeared featureless under optical microscopy.

**Figure 4.**
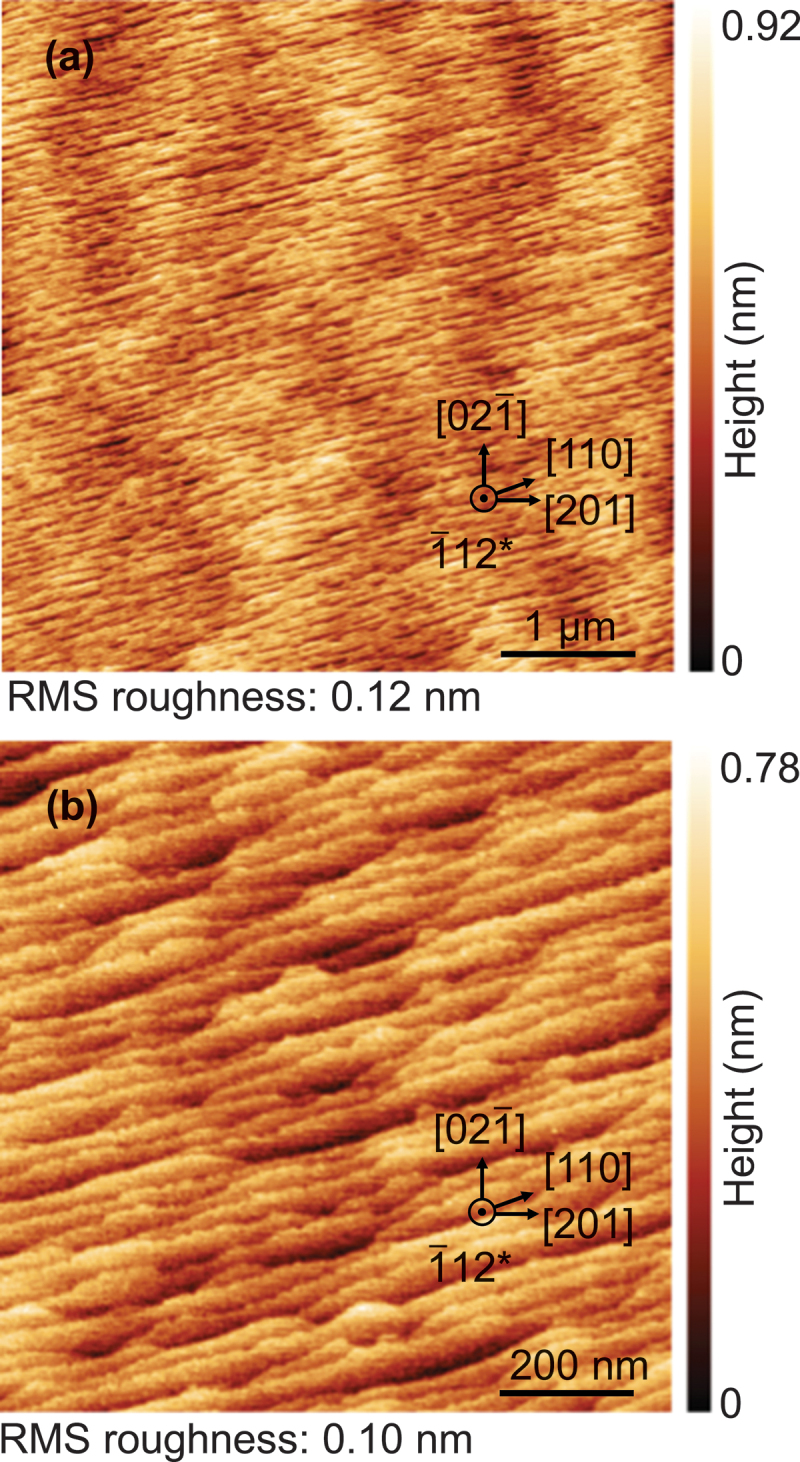
Atomic force microscopy images of a flat surface region of the ($\overset{ˉ}{1}$12) epilayer with scan areas of (a) 5 μm × 5 μm and (b) 1 μm × 1 μm.

The surface morphology of the flat region was further investigated by AFM, as shown in Figure 4. The surface was revealed to consist of stairs-like step-and-terrace structures, providing an ideal surface for various applications. Considering the monolayer height of 0.21 nm (Figure 1), the steps were composed of monolayer- and bilayer-height steps, which correspond to dark and darker contrasts in the images. The root-mean-square (RMS) roughness values for 5 μm × 5 μm and 1 μm × 1 μm scan areas were 0.12 and 0.10 nm, respectively. These RMS values are among the lowest reported for homoepitaxial β-Ga_2_O_3_ surfaces across various substrate orientations and growth techniques [42]. Notably, such a flat epitaxial surface on the ($\overset{ˉ}{1}$12) plane does not require a post-growth CMP process and is readily applicable to device fabrication. This represents a significant advantage over (001) epilayers, for which post-growth CMP is required to remove approximately half of the layer thickness [40], even when the lower growth rate of the ($\overset{ˉ}{1}$12) plane relative to the (001) plane is taken into account.

**Figure 5.**
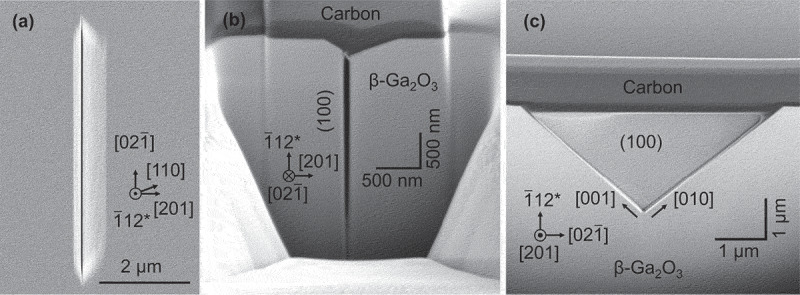
**(*Two columns)*** (a) Plan-view and (b) and (c) 54°-tilted cross-sectional scanning electron microscopy images of a slit-like pit observed on the ($\overset{ˉ}{1}$12) epilayer. The exposed cross sections were perpendicular to the $[02\overset{ˉ}{1}]$ direction in (b) and parallel to the $\left[02\overset{ˉ}{1}\right]$ direction in (c).

On the other hand, in order to examine the origin of the linear pits, they were investigated from multiple viewing directions using SEM, as shown in Figure 5. Figure 5(a–c) correspond to a top view, a 54°-tilted cross-sectional view along $\left[0\overset{ˉ}{2}1\right]$, and a 54°-tilted cross-sectional view along [201], respectively. Note that the FIB vertical cross-sectioning in Figure 5(b,c) was performed in planes perpendicular and parallel to the $\left[02\overset{ˉ}{1}\right]$-elongated linear pits, which means that the cross sections were perpendicular and parallel to the (100) plane, respectively. From these images, the linear pit was revealed to be a thin slit sandwiched between vertically aligned (100) facets and to have an isosceles triangular cross-sectional profile with a right-angled vertex pointing downward. Although we could not unambiguously identify the crystallographic planes forming the two equal-length sides of the isosceles triangular profile due to the very small slit width, they are facets parallel to the [001] and [010] directions. This inverted triangular shape indicates that the slit originated at the vertex and grew upward along the (100) facets, with its sides propagating along the [001] and [010] directions. The vertex depth was 2.6 μm, which closely agrees with the 2.8-μm thickness measured by SIMS. This agreement indicates that the pits originated at the layer/substrate interface, which likely explains why the observed linear pits on the surface have the same length. It should be noted that such (100)-defined slit-like pits were also found in homoepitaxial layers grown on (011) β-Ga_2_O_3_ substrates using the same HVPE system [15], though the pits were tilted with respect to the surface normal.

**Figure 6.**
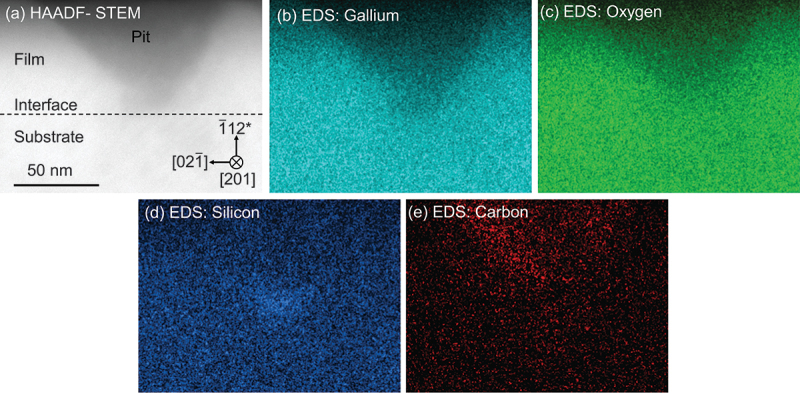
**(*Two columns)*** (a) Cross-sectional high-angle annular dark field scanning transmission electron microscopy (HAADF-STEM) image and (b)–(e) energy-dispersive X-ray spectroscopy (EDS) elemental maps of Ga, O, Si, and C, respectively, in the vicinity of the pit-generation region.

To identify the origin of the pit formation, cross-sectional STEM-EDS was performed in the vicinity of the vertex of the pit at the interface, as shown in Figure 6. Figure 6(a) shows a high-angle annular dark field (HAADF) STEM image, and Figure 6(b–e) show EDS maps of Ga, O, Si, and C for the corresponding scan area, respectively. Note that the TEM support mesh contains Si; therefore, the background Si signal is slightly higher than that of the other elements. The reduced Ga and O signals and the enhanced C signal in the pit region are consistent with the absence of β-Ga_2_O_3_ and the presence of a C deposit, used as a protective layer for FIB milling, respectively. In contrast, the enhanced Si signal at the pit vertex suggests the presence of unintentional SiO_2_ nanostructures, which likely originate from quartz parts in the reactor, such as the quartz substrate holder and quartz tube. Because SiO_2_ can act as a mask for selective-area growth for β-Ga_2_O_3_ [47], its presence at the interface reasonably accounts for the initiation of pit formation. Other masking species may also contribute, such as CMP-induced surface defects or deposited β-Ga_2_O_3_ particles generated during the initial stage of growth. In any case, these pits are not intrinsic to the ($\overset{ˉ}{1}$12) orientation but are related to the substrate surface condition and/or the early growth process, and thus should be mitigated by improving pre-growth surface treatments and optimizing the initial growth conditions.

**Figure 7.**
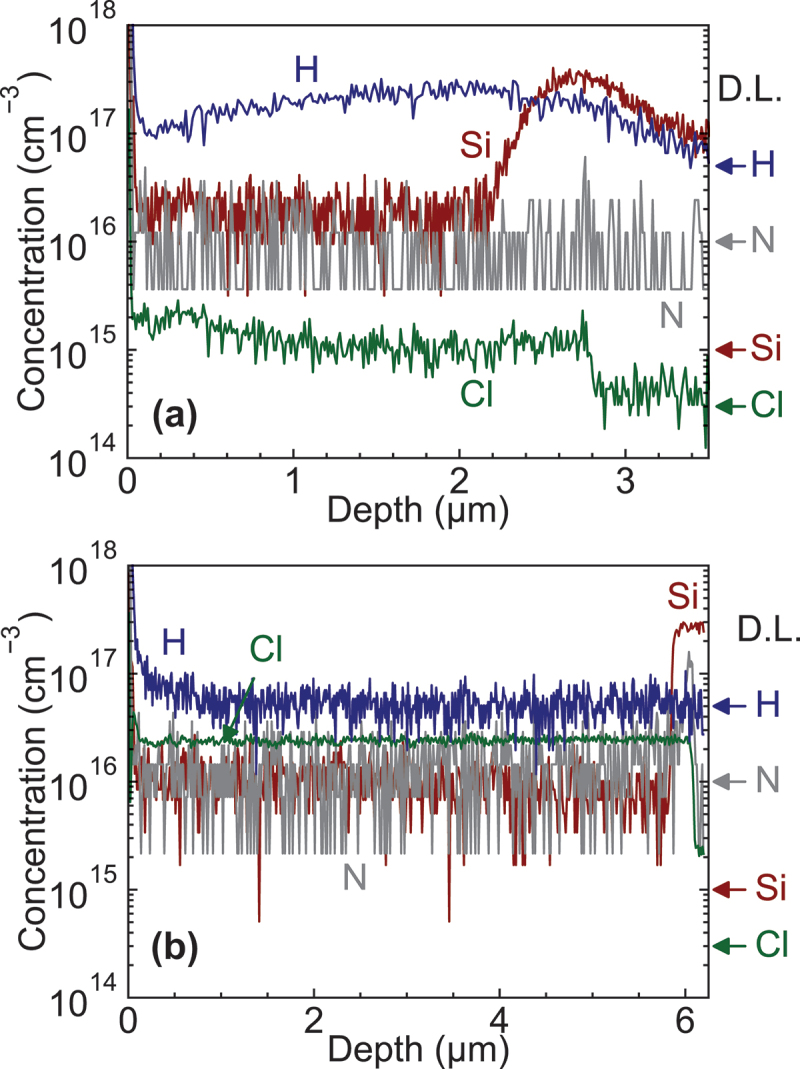
Depth profile of impurities in (a) the ($\overset{ˉ}{1}$12) and (b) the (001) epilayers obtained by secondary ion mass spectroscopy. Arrows indicate detection limits.

Finally, the impurity concentrations in the ($\overset{ˉ}{1}$12) and (001) layers were compared. Figure 7(a,b) show depth profiles of atomic concentrations of unintentionally doped Si, Cl, N, and H in the homoepitaxial layers grown on the ($\overset{ˉ}{1}$12) and (001) substrates, respectively. Here, we particularly focus on Si and Cl as they are major donor impurities in HCl-based HVPE. The Si concentrations were comparable, with values of 2 × 10^16^ cm^−3^ and 1 × 10^16^ cm^−3^ in the ($\overset{ˉ}{1}$12) and (001) layers, respectively. The incorporation of Si is attributable to the environment in the growth zone, where the hydrogen and quartz components coexist [48], because reactive hydrogen slightly etches the quartz. However, this etching reaction can be prevented by replacing quartz components with quartz-free alternatives. On the other hand, the incorporation of Cl cannot be avoided as long as GaCl_*x*_ species are used as precursors and is therefore inevitable in HVPE. Nevertheless, the Cl concentration of the ($\overset{ˉ}{1}$12) layer was as small as 1 × 10^15^ cm^−3^, which was significantly lower than that of the (001) layer (2 × 10^16^ cm^−3^). This non-negligible orientation dependence of Cl incorporation is likely related to differences in surface bonding configurations between these two crystal planes, although the mechanism remains to be clarified in the future work. The difference in growth rate may also affect Cl incorporation because Cl could be incorporated into the growing layer before sufficient desorption occurs at the grown surface. In our previous experiments, a (001) homoepitaxial layer grown at a lower growth rate of 8.4 μm h^−1^ still showed a relatively high Cl concentration of 4.1 × 10^15^ cm^−3^. Although further systematic studies are required to separate the effects of growth rate and surface orientation, this result suggests that the pronounced reduction in Cl concentration for the ($\overset{ˉ}{1}$12) layer cannot be explained by the growth rate alone and is likely associated with the crystallographic orientation of the growth surface. It should be noted that Si concentrations were comparable between the ($\overset{ˉ}{1}$12) and (001) orientations, whereas Cl concentrations were significantly different. One possible explanation is that Si and Cl are incorporated into different lattice sites, namely Ga and O sites, respectively, and are therefore affected by different bonding states even for the same substrate orientation. In addition, Si may be more readily incorporated into the lattice than Cl because the Si–O bond is stronger than the Cl–Ga bond. This difference in bonding strength could result in weaker and stronger orientation dependence for Si and Cl incorporation, respectively. The Cl concentration of the $(\overset{ˉ}{1}$12) plane, in the low 10^15^ cm^−3^ range, was sufficiently small, given that the doping control required for vertical power devices is typically in the low 10^16^ cm^−3^ range. Therefore, the $(\overset{ˉ}{1}$12) substrate is attractive not only for its excellent epitaxial surface morphology but also for its sufficiently low Cl incorporation.

## Hcl-gas etching on ($\overset{ˉ}{1}$12) β-Ga_2_O_3_

### Experimental methods

Prior to the etching process, a SiO_2_ mask with etching windows was fabricated on a ($\overset{ˉ}{1}$12) substrate by plasma-enhanced CVD using tetraethyl orthosilicate and O_2_ precursors, followed by laser lithography and capacitively coupled plasma reactive-ion etching with a CHF_3_/N_2_ gas mixture. To investigate the HCl gas etching behavior, wagon-wheel and stripe-shaped windows were employed. The wagon-wheel pattern consists of 72 linear windows arranged at 5° intervals, starting from the [201] direction, which is perpendicular to (100). The length and width of each window were 50 and 0.7 μm, respectively. The stripe pattern consists of linear windows aligned along the $\left[02\overset{ˉ}{1}\right]$ direction, which is parallel to (100). The window and mask widths were 0.8 and 1.2 μm, respectively.

Selective-area HCl-gas etching was performed on the SiO_2_-masked ($\overset{ˉ}{1}$12) substrate in the same HVPE system that has also been employed in our previous gas etching experiments on (100), (010), (001), ($\overset{ˉ}{1}$02), and (011)-oriented β-Ga_2_O_3_ substrates [44,49–55]. Etching was carried out by supplying HCl gas to the substrate in the growth zone (used here as the etching zone) heated to 1038°C. The HCl gas was carried by N_2_ gas under atmospheric pressure, while the total gas flow rate was kept at 8 slm. The HCl partial pressure was 62.5 Pa. The etching time was 10 min.

The resulting etched structures were examined using SEM and FIB–SEM.

### Results and discussion

**Figure 8.**
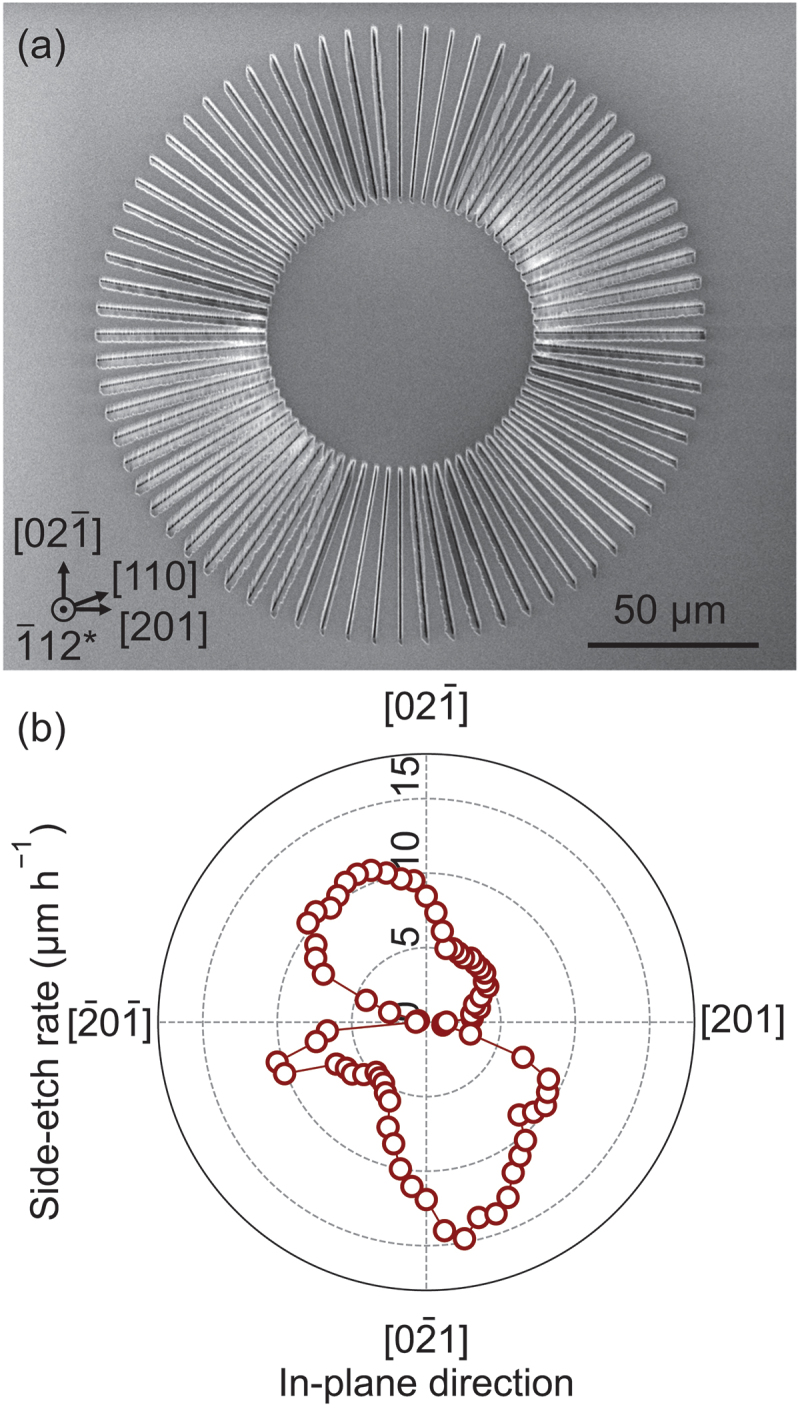
Summary of side-etching characterization for trenches etched beneath the wagon-wheel window pattern on the ($\overset{ˉ}{1}$12) substrate. (a) Plan-view scanning electron microscopy image. (b) Polar plot of the side-etch rates extracted from the trenches shown in (a).

Anisotropy of in-plane side etching was first examined by measuring the side-etch rates of the wagon-wheel-patterned etched trenches, as shown in Figure 8. The measured side-etch rates from the top-view SEM image (Figure 8(a)) were summarized in a polar plot (Figure 8(b)). Note that the side-etch rate was calculated from the side-etch length of the trenches, defined as the spacing between the etching front and the mask edge, which could be measured by SEM observation from the surface normal at an acceleration voltage (*V*_acc_) of 10 kV [50]. The side etching was remarkably suppressed for the $\left[02\overset{ˉ}{1}\right]$-oriented trenches; that is, side etching along the [201] and $[\overset{ˉ}{2}$0$\overset{ˉ}{1}]$ directions was minimized, suggesting the development of high etch-resistant (100) sidewall facets with the lowest surface energy density [41]. In addition, the shape of the polar plot for the ($\overset{ˉ}{1}$12) substrate exhibited an approximate twofold in-plane rotational symmetry, which is very similar to that for the (011) substrate [55]. The similarity likely reflects the close crystallographic relationship between the ($\overset{ˉ}{1}$12) and (011) planes, as described earlier.

**Figure 9.**
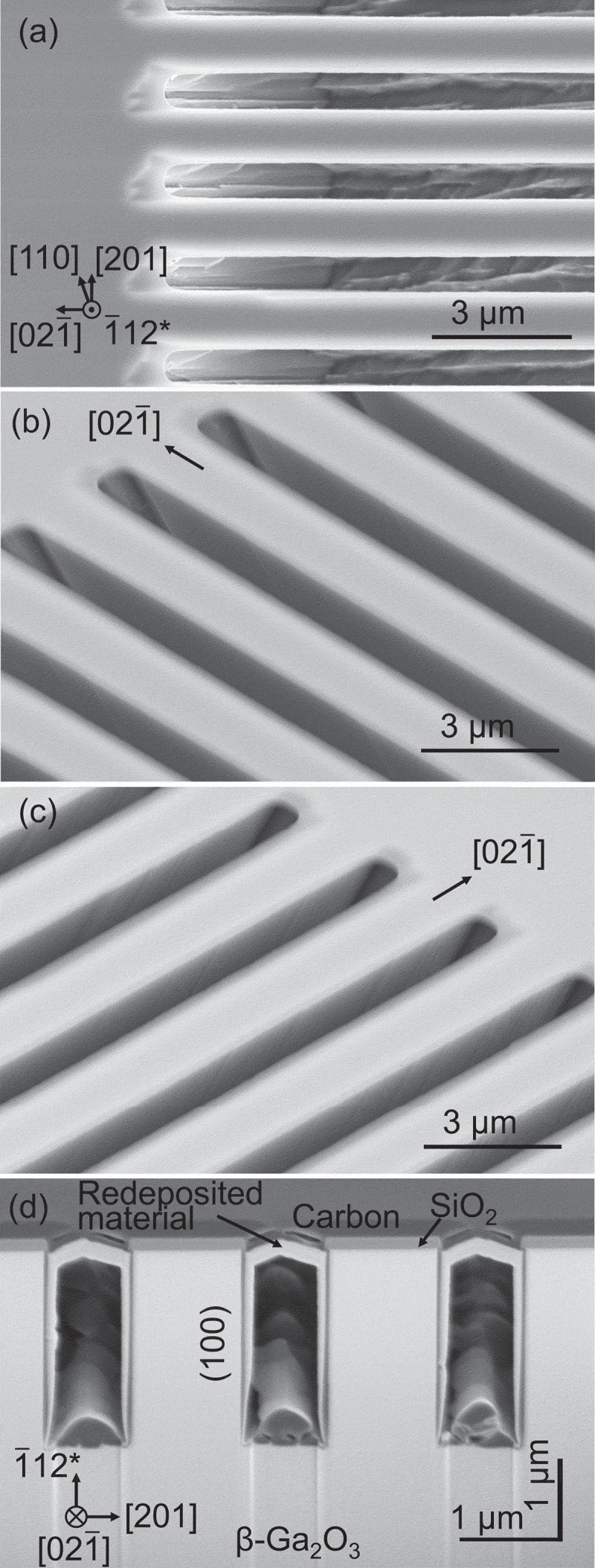
Summary of scanning electron microscopy observations of the trench array aligned along the $\left[02\overset{ˉ}{1}\right]$ direction on the ($\overset{ˉ}{1}$12) substrate. (a) Plan-view image. (b) and (c) 54°-tilted-view images taken from different in-plane directions. (d) 54°-tilted-view image of the cross-section perpendicular to the $\left[02\overset{ˉ}{1}\right]$ direction.

Then, a trench array aligned along the $\left[02\overset{ˉ}{1}\right]$ direction, which exhibited the minimum lateral etching, was observed using SEM, as shown in Figure 9. Figure 9(a–c) show SEM images near the trench ends, acquired in plan-view and tilted-view geometries with the tilt axis oriented along two different in-plane directions, respectively. Note that these images were taken at *V*_acc_ of 10 kV to clearly reveal the etching fronts. Figure 9(d) shows a cross-sectional tilted-view SEM image of the corresponding trenches near the center of the array; the cross section was taken perpendicular to the trenches, i.e. along the (100) plane. In this case, *V*_acc_ was set to 2 kV to enhance material contrast. These images collectively revealed that the trench profiles consisted of vertical, extremely flat (100)-faceted sidewalls and a relatively rough ($\overset{ˉ}{1}$ 12) bottom surface. The side-etch length and height of the (100) sidewalls were 0.04–0.05 and 2.43–2.48 μm, respectively, yielding a vertical-to-lateral etch-rate ratio of 47–55. This aspect ratio regarding the (100) sidewalls is higher than those of HCl-gas-etched vertical trenches on (010) substrates (11–14) [49] and ($\overset{ˉ}{1}$02) substrates (7.9–11.2) [54] formed under the same etching conditions, representing the highest value among the major oxygen-sublattice-derived substrate orientations that enable vertical trench formation [43]. This high anisotropy also enables precise etching that faithfully reproduces the mask pattern. Although the bottom surface roughness may be a concern, it is known to be mitigated by lowering the etching temperature [53,56]. Furthermore, unlike conventional plasma-based dry etching, gas-phase etching such as HCl-gas etching introduces no plasma-induced damage to the processed surfaces and is therefore well suited for device applications. Therefore, the ($\overset{ˉ}{1}$12) orientation is particularly beneficial as a platform for trench/fin-based devices that require vertical, smooth sidewall structures free of plasma damage.

## Conclusion

We proposed the $(\overset{ˉ}{1}12)$ orientation as a suitable crystallographic plane for homoepitaxy and selective-area gas etching of β-Ga_2_O_3_, and demonstrated its advantages for both growth and etching using our HCl-based HVPE system. In homoepitaxy, we achieved twin-free growth without miscut control and an atomically flat surface characterized by a well-ordered step-and-terrace morphology. Furthermore, the Cl impurity incorporation attributed to HVPE was sufficiently low, in the low-10^15^ cm^−3^ range. In gas etching, we confirmed that anisotropic etching with a high vertical-to-lateral etch-rate ratio of approximately 50 was possible owing to the formation of vertical, extremely flat (100)-faceted sidewalls when the etching mask windows were aligned along the $\left[02\overset{ˉ}{1}\right]$ direction. Taken together, these growth and etching results underscore the potential of the $\left(\overset{ˉ}{1}12\right)$ plane for β-Ga_2_O_3_ power devices that require high-purity, smooth homoepitaxial layers, and high-aspect-ratio trench/fin structures with vertical and flat sidewalls without plasma damage.
